# Body image in children with gender incongruence

**DOI:** 10.1177/13591045211000797

**Published:** 2021-03-24

**Authors:** Anouk Verveen, Baudewijntje PC Kreukels, Nastasja M de Graaf, Thomas D Steensma

**Affiliations:** 1 Center of Expertise on Gender Dysphoria, Amsterdam UMC, location VUmc, Amsterdam, The Netherlands; 2 Department of Medical Psychology, Amsterdam UMC, location VUmc, The Netherlands

**Keywords:** Children, gender incongruence, body image, body satisfaction

## Abstract

**Background:**

In the DSM-5 diagnosis of childhood Gender Dysphoria, two of the eight criteria focus on body satisfaction of the child. Nevertheless, this subject is understudied. This study aims to describe the body image of children with gender incongruence (GI) in relation to birth assigned sex and the intensity of GI.

**Method:**

Self-report and parent-report measures on body satisfaction and gender incongruence were obtained from 207 children (<12 years) who were referred to the Center of Expertise on Gender Dysphoria at the Amsterdam University Medical Centers, location VUmc, between 2010 and 2016. First, a general description of body satisfaction in children who took part in this study is provided. Secondly, body image of birth assigned boys and girls are compared using chi-square tests and univariate ANCOVA’s. Thirdly, the association between intensity of GI and body image is examined using multiple linear regression analyses.

**Results:**

Of the 207 children with GI, 50% reported dissatisfaction with their gender-specific characteristics. Overall, children were less dissatisfied with their neutral body characteristics. Birth assigned girls report greater dissatisfaction with their body characteristics than birth assigned boys. Intensity of GI was significantly related to satisfaction with gender specific body characteristics where a greater intensity of GI relates to more body dissatisfaction.

**Conclusion:**

Mental health practitioners should be aware of the diversity in body dissatisfaction in this group. Furthermore, evaluation of body image should be an important topic in the counseling of these children. Future research should focus on the relation of body dissatisfaction and the development of gender incongruent feelings in children with GI.

## Introduction

Over the years, the attention for gender incongruence (GI) in children and youth has increased. Whilst knowledge on body image in adults and adolescents struggling with gender identity issues is increasing, this knowledge is scarce in children with gender incongruence. To our knowledge, this study is the first to present on body image and satisfaction of children (<12 years) with gender incongruence.

Sex is usually identified by the external genitalia and serves as the basis for the assignment of legal ‘gender’ (Meyer-Bahlburg, 2010). Gender is how one identifies, male, female or between or outside that binary (Richards et al., 2016). For some children, their gender identity is not congruent to their birth assigned sex. These children experience gender incongruence (GI) (Meyer-Bahlburg, 2010). When there is an incongruence between birth assigned sex and experienced gender, together with significant feelings of distress, children may fulfil the criteria of the DSM-5 diagnosis of Gender Dysphoria (GD) (American Psychological Association, 2012; Lopez et al., 2016). In this study, we use the term gender incongruence when referring to the total group of referred children to our clinic. We use the term gender dysphoria when referring to children who received a formal diagnosis of Gender Dysphoria in childhood as described in the DSM-5. Birth assigned boys will be referred to as AMAB (assigned male at birth), birth assigned girls as AFAB (assigned female at birth)^1^.

The diagnosis of GD is based on criteria in the Diagnostic and Statistical Manual of Mental Disorders (DSM). These DSM-5 criteria for childhood GD are mostly focused on behavior, however, two criteria are concerning the body image of the child. These criteria are: *A strong dislike of one’s sexual anatomy* and *A strong desire for the physical sex characteristics that match one’s experienced gender* (American Psychiatric Association, 2013). To be diagnosed with childhood GD, six of the eight diagnostic criteria have to be present. Therefore, neither of the criteria on body image have to be present for a child to be diagnosed with GD, which raises the question to what extent body dissatisfaction is present in the population of children diagnosed with GD and referred to gender identity clinics.

Body image describes a multifaceted construct, which refers to the perceptions, thoughts, and feelings individuals have about their body and bodily experiences (Cash, 2012; Lindgren & Pauly, 1975). Previous studies on adults and adolescents with GI have shown that individuals with GI report lower levels of satisfaction with their overall appearance compared to individuals without GI (Becker et al., 2016, 2018; Pauly & Lindgren, 1977; Witcomb et al., 2015). To our knowledge, no systematic description of body image in pre-pubertal children with GI is available.

An explanation for the low body satisfaction in individuals with GI may be the visible dissonance between the birth assigned sex and the experienced gender. This is enforced by findings that gender affirming hormone treatment, with effects on sex-specific body characteristics, has a positive effect on body satisfaction (Becker et al., 2016; Fisher et al., 2014), while puberty blockers do not have an effect (De Vries et al., 2011, 2014). Further along the process of gender affirming hormone treatment, the body image of the individual improves with the increasing resemblance of their body characteristics to their experienced gender (Becker et al., 2016). Social transitioning also has a positive effect on body satisfaction in adults (Van de Grift et al., 2016b).

For the general population, body dissatisfaction of adolescents increases during puberty, and then decreases with age in adulthood (Tiggemann & McCourt, 2013; Truby & Paxton, 2002). Very young children (<5 years) rarely experience hatred or aversion towards their own bodies and body satisfaction of pre-pubertal children is higher than that of pubertal children (Folk et al., 1993). It is known that childhood gender nonconformity is related to body dissatisfaction and that there is a relation between body image and experienced masculinity and femininity (Borchert & Heinberg, 1996). Masculinity and femininity are general constructs that relate to an individual’s gender identity. It has been observed that children with GI start to feel more uncomfortable with their bodies during and after puberty, when feminization (in AFAB) or masculinization (in AMAB) leads to a more sex-specific appearance and an increased incongruence between birth assigned sex and experienced gender (e.g. Pollock & Eyre, 2012; Van de Grift et al., 2016a).

There are birth assigned sex differences in body satisfaction. In the general population, AFAB aged 9 to 11 experience less body satisfaction compared to AMAB of the same age (Xanthopoulos et al., 2011). Adult AFAB are generally less satisfied with their body than AMAB (Grogan, 2017). Similarly, trans women (AMAB) have lower body satisfaction than trans men (AFAB) (e.g. Van de Grift et al., 2016b; Vocks et al., 2009). It is yet unknown if there are differences in body satisfaction between pre-pubertal AFAB and AMAB.

It is important to gain knowledge on body satisfaction of children as the level of body satisfaction in children in the general population appears to be a predictor of body satisfaction in adulthood (Smolak, 2004). Furthermore, body satisfaction is related to psychological well-being of adults with GI. A negative body image is associated with lower self-esteem and poorer social functioning, which is associated with a lower quality of life (Van de Grift et al., 2016b). Insight into the body satisfaction of children can have potential value in GI counseling.

This study aims to provide a better understanding of childhood GI in relation to body image. The main research objectives are (1) to describe body (dis)satisfaction in children with GI; (2) to examine birth assigned sex differences; and (3) to examine the association between body satisfaction and intensity of GI. We hypothesize that children with GI have a low level of satisfaction with overall body appearance. We expect they will be least satisfied with sex characteristics. AFAB will have a lower level of body satisfaction than AMAB. Furthermore, it is expected that the children with more intense GI will have a more negative body image.

## Methods

### Participants and procedure

The participants in this study consisted of an initial sample of 289 children who were referred to the Center of Expertise on Gender Dysphoria at the Amsterdam University Medical Center (Amsterdam UMC), location VUmc, between 2010 and 2016. These children followed the child treatment pathway at the center, which indicates that these parcticipants were not yet in puberty. The child treatment pathway generally includes children younger than 12 years at first assessment in the clinic. For inclusion the diagnostic phase and relevant measures that were used in this study had to have been completed. If the diagnostic phase was not completed or if there was incomplete data, the child was excluded from the study. Finally, 207 children were included, of which 83 AMAB and 124 AFAB.

We checked for differences in the excluded and included group. The excluded group of children had significantly more AMAB’s (56.1% (46)) compared to AFAB’s (43.9% (36)), (χ^2^ (1) =  6.085, *p* = .014). The excluded children also were significantly younger (8.03 (2.26)) than the included children (8.66 (1.62)), (*t* (115) = −2.279, *p* = .025). There was no significant difference in IQ.

Official approval of this study by our medical ethical commission was not required as the Medical Research Involving Human Subjects Act (WMO) does not apply to the study. Informed consent procedure and data handling was done following the Guidelines for Good Clinical Research Practice. For this study the parents provided informed consent for the use of clinical information of the child in clinical evaluation and exploration studies.

### Design and measures

#### Demographic information

Background and demographic information included: age measured in years, birth assigned sex, parent’s marital status, IQ, and birth weight (in grams). Parent’s marital status was classified as ‘living with two parents’ (biological parents, parent with step-parent) and ‘other’ (e.g. single parent, adopted, foster care). IQ was assessed using the Dutch versions of the Wechsler Preschool and Primary Scale of Intelligence or the Wechsler Intelligence Scale for Children (Wechsler, 1989, 1991).

### Body image and satisfaction

Three questionnaires on body satisfaction for children were part of the diagnostic evaluation of these children, and were therefore used. These were the Physical attraction scale (PAS), the Physical Features Satisfaction Scale (PFSS) and the gender specific factor of the Anatomic Satisfaction Scale (GS-ASS). These scales were developed by Susan L. Lambert in 2009 and described in her unpublished thesis. The body image questionnaires used in the study can be found in appendix A-C. For all questionnaires a Dutch translation was used. The GS-ASS has different versions for AMAB and AFAB, based on birth assigned sex. With the exception of pronouns, the PAS, PFSS, GII, and GIQ questionnaires have similar versions for AMAB and AFAB.

*The Physical Attraction Scale (PAS)* is a self-report measure where the child is asked to assess their self-perceived physical attractiveness while looking in a mirror. Five items assessed the self-perception of attractiveness (cute, pretty, ugly, beautiful, and handsome). For each item there were five response categories ranging from ‘not at all’ (0) to ‘very much’ (4). The item ugly was reverse scored so that a higher total score corresponded to a higher level of self-perceived attractiveness (range, 0–20). The scale had a moderate Cronbach’s alpha of .677.

*The Physical Features Satisfaction Scale (PFSS)* is a self-report measure that was based on the Body Image scale developed by Lindgren and Pauly (1975) and is used to evaluate specific domains of body satisfaction in the child. Eight items assessed satisfaction with different body parts (nose, hips, chin, legs, hands, arms, chest, and face). For each item there were five response categories ranging from ‘not at all’ (0) to ‘very much’ (4). A higher total score corresponded to a higher level of satisfaction with body parts (range, 0–32) (Lambert, 2009). The scale had a moderate Cronbach’s alpha of .789.

*The Gender Specific factor of the Anatomic Satisfaction Scale (GS-ASS)* is a parent-report measure derived from the Anatomic Satisfaction Scale (ASS) (Zucker, 2010). Lambert assessed the initial 31 item questionnaire in gender incongruent children. Based on a factor analysis, Lambert created a 6 item scale specifically focused on gender specific body satisfaction questions, which was used in this study. The questions focus on primary sex characteristics (penis, vagina), future secondary sex characteristics (breasts), face, and general appearance. The parents had to respond whether the question referred to their child (yes, sometimes, no). A lower total score corresponded to more dissatisfaction (range, 6–18) (Lambert, 2009). The scale had a moderate Cronbach’s alpha of .667.

For all three measures of body image a threshold score was determined. This range was determined based on the neutral total score for each questionnaire. On the PAS and PFSS each item could be scored on a scale from 0 (not at all) to 4 (very much). A score of 2 (sometimes) could be seen as neutral. As the PAS consists of five items, the threshold score of body satisfaction was determined to be a total score of 10. Children with a score of 10 or above were considered to be generally satisfied with their body. For the PFSS, eight items, a total score <16 was determined to be below the threshold of satisfaction. The GS-ASS consists of six items, scored from 1 (yes) to 3 (no). A score of 2 (sometimes) was neutral. For the GS-ASS a total score <12 was determined to be below the threshold of body satisfaction and therefore in the clinical range of body dissatisfaction.

### Gender incongruence

Two questionnaires were used to determine intensity of gender incongruent/dysphoric feelings.

*The Gender Identity Interview (GII)* is a self-report questionnaire that includes items on affective and cognitive gender confusion. Affective gender confusion is the child’s desire to be a member of the other sex. Cognitive gender confusion is when a child mislabels his or her gender, or shows a lack of cognitive gender constancy over time (Zucker et al., 1993). The GII consists of 12 questions which are scored on a 3-point scale ranging from 0 to 2. A 0 was assigned if the child answered a factual question correctly or gave a stereotypic response. A 1 was assigned for ambiguous or intermediate responses such as ‘I don’t know’ to the question. ‘Do you think it is better to be a boy or a girl?’ A 2 was assigned to responses that were in line with their desired gender and without ambiguity. Children who gave more answers in line with their desired gender identity have a higher score. This correlates to more GI (Zucker et al., 1993). The Dutch translation of the GII was used, which was validated by Wallien et al. (2009).

*The Gender Identity Questionnaire (GIQ)* is a parent-report questionnaire, that is used in the assessment of children with potential problems in their gender identity development (Johnson et al., 2004). The questionnaire consists of 16 items that cover a range of sex-typed behaviors. Two of these items (15 and 16) focus on (dis)like of sexual anatomy, therefore these items were excluded to ensure independence of outcome measures. Furthermore item 8 was excluded in line with the results of validity studies (Cohen-Kettenis et al., 2006; Johnson et al., 2004). As a result, this study used a 13-item GIQ. Items are recoded according to standard scoring rules, lower scores reflect more cross-gendered behavior. Gender referred children have a significantly lower score than controls (Johnson et al., 2004).

### Statistical analysis

Chi-square tests and *t*-tests were performed on nominal variables to determine between-group differences. Overall scores and scores on individual items of PAS, PFSS, and GS-ASS were compared by birth assigned sex by means of independent *t*-tests and one-way ANOVAs. ANCOVA analyses were performed to control for the covariate age. For analyses on individual items, Bonferroni corrections were done. Multiple linear regression analyses were performed on the measures for intensity of GI, sex, and age in relation to the body satisfaction measures. Assumptions were checked before running the analyses.

Statistical analyses were performed with IBM SPSS Statistics version 24. An alpha (α) of .05 was considered significant.

## Results

### Sample characteristics

The children in the study were between the ages of 5 and 12 years at clinical entry. Demographic characteristics of the study population are presented in Table 1. Independent *t*-tests were performed on the demographic data to test for any group differences on the descriptive variables. AFAB were significantly older than AMAB (*t* (205) = −2.689, *p* = .008). No significant sex differences were observed for marital status, IQ, birth weight, GIQ, or GII.

**Table 1.** Sample characteristics.

	Total group (*n* = 207)	AMAB (*n* = 83)	AFAB (*n* = 124)
Age at assessment (*M, SD*)	8.66 (1.622)	8.29 (1.693)*	8.90 (1.532)*
Marital status (%, *N*)			
Living with two parents	80.4 (160)	79.7 (63)	80.8 (97)
Other	19.6 (39)	20.3 (16)	19.2 (23)
IQ (*M, SD*)	100.77 (15.972)	102.25 (16.080)	100.53 (13.622)
Birth weight (*M, SD*)	3465.54 (798.412)	3531.11 (1044.301)	3427.14 (612.866)
GIQ mean (*M, SD*)^a^	1.9084 (0.45813)	1.8606 (0.51468)	1.9402 (0.41576)
GII sum (*M, SD*)^b^	14.83 (5.640)	13.78 (6.394)	15.62 (4.898)

a GIQ: Gender Identity Questionnaire: corrected for the two body image items. Range, 1 to 5.

b GII: Gender Identity Interview. Range, 1 to 24.

* *p* < .05.

### Body image and sex differences

Table 2 shows the means and standard deviations of the measures of body satisfaction, also separately for birth assigned sex. Table 3 shows the means and standard deviations of the individual items of body satisfaction.

**Table 2.** Ratings of body satisfaction, split by birth assigned sex.

Variables		Total score			% in the clinical range (*n*)		
		Total (*n* = 172)	AMAB (*n* = 65)	AFAB (*n* = 107)	Total (*n* = 172)	AMAB (*n* = 65)	AFAB (*n* = 107)
PAS sum^a^	*M*	12.77	13.88*	12.09*	19.0 (32)	10.9 (7)*	24.0 (25)*
	*SD*	3.881	3.383	4.024			
PFSS sum^b^	*M*	24.90	24.98	24.85	4.1 (7)	3.1 (2)	4.7 (5)
	*SD*	5.820	5.296	6.140			
GS-ASS sum^c^	*M*	11.68	12.15	11.42	49.4 (83)	41.9 (26)	46.2 (57)
	*SD*	2.631	3.141	2.255			

a PAS: Physical Attraction Scale (*N* = 168), range, 0 to 20.

b PFSS: Physical Features Satisfaction Scale (*N* = 172), range, 0 to 32.

c GS-ASS: The Gender Specific Factor of the Anatomic Satisfaction Scale (*N* = 168), range, 6 to 18.

* *p* < .05.

**Table 3.** Ratings on the individual items of the body satisfaction scales for the total group and for birth assigned sex.

PAS^a^			Total (*n* = 168)	AMAB (*n* = 64)	AFAB (*n* = 104)
1	I am cute	*M*	1.85	2.19*	1.63*
		*SD*	1.250	1.149	1.267
2	I am pretty	*M*	2.55	2.64	2.50
		*SD*	1.215	1.211	1.221
3	I am ugly	*M*	3.41	3.49	3.36
	*Reverse scored*	*SD*	0.931	0.819	0.997
4	I am beautiful	*M*	2.86	3.24*	2.62*
		*SD*	1.202	1.024	1.251
5	I am handsome	*M*	2.13	2.39	1.96
		*SD*	1.267	1.214	1.277
PFSS^a^			Total (*n* = 172)	AMAB (*n* = 65)	AFAB (*n* = 107)
1	Nose	*M*	3.13	3.09	3.15
		*SD*	1.128	1.203	1.084
2	Hips	*M*	2.92	3.12	2.80
		*SD*	1.297	1.225	1.331
3	Chin	*M*	2.94	2.58	3.16
		*SD*	1.242	1.394	1.087
4	Legs	*M*	3.06	3.16	3.00
		*SD*	1.246	1.136	1.311
5	Hands	*M*	3.40	3.30	3.46
		*SD*	0.987	0.960	1.003
6	Arms	*M*	3.47	3.39	3.51
		*SD*	0.925	0.909	0.935
7	Chest	*M*	2.53	2.69	2.44
		*SD*	1.387	1.310	1.429
8	Face	*M*	3.36	3.40	3.34
		*SD*	0.923	0.880	0.951
GS-ASS^b^			Total (*n* = 168)	AMAB (*n* = 62)	AFAB (*n* = 106)
1	Does ___ say that (s)he would like a vagina/penis?	*M*	1.83	2.11	1.67*
		*SD*	0.822	0.851	0.761
2	Does ___ say that (s)he would like breasts/no breasts?	*M*	1.63	1.97	1.44*
		*SD*	0.814	0.849	0.729
3	Does ___ say that (s)he wished he looked like a girl/boy?	*M*	1.13	1.23	1.07
		*SD*	0.401	0.493	0.327
4	Does ___ say that (s)he wants to get rid of his penis/vagina?	*M*	2.21	2.10	2.27
		*SD*	0.821	0.804	0.827
5	Does ___ say that (s)he wishes his/her face were prettier?	*M*	2.74	2.60	2.82*
		*SD*	0.599	0.712	0.508
6	Does ___ pretend that (s)he has breasts/a penis?	*M*	2.15	2.15	2.15
		*SD*	0.741	0.721	0.756

a PAS: Physical Attraction Scale.

b PFSS: Physical Features Satisfaction Scale.

c GS-ASS: The Gender Specific Factor of the Anatomic Satisfaction Scale.

* *p* < .05.

The PAS was completed by 168 children. Of these children 19.0% scored in the clinical range of dissatisfaction. There was a significant difference in the scores for AFAB and AMAB on total score of the PAS (*t* (166) = 2,967, *p* = .003). The ANCOVA showed that the covariate age significantly affects the scores of the PAS (*F*(1,165) = 5,704, *p* = .018). Including age as a covariate, the scores on the PAS still significantly differed for sex (*F*(1, 165) = 7.988, *p* = .005). AFAB scored significantly lower on the PAS than AMAB. Chi-square tests similarly showed there was a significantly higher percentage of AFAB (24.0%) that scored in the clinical range of dissatisfaction compared to AMAB (χ^2^ (1) = 4.41, *p* = .036). An ANCOVA with the covariate age was used to examine group differences between scores on the individual items of the PAS, which are shown in Table 3. After Bonferroni correction, there was a significant sex difference for the items cute (*F*(1, 171) = 12.39, *p* = .005) and beautiful (*F*(1, 170) = 13.98, *p* = .001). AFAB scored lower than AMAB on all individual items of the PAS.

The children had a mean score of 24.90 on the PFSS. ANCOVA revealed no significant effect of sex on the total score of the PFSS (*N* = 172), even when corrections for age were made. Of the individual items, only the item ‘chin’ (*F*(1, 172) = 13.67, *p* = .003) was significantly different for AMAB and AFAB, where AMAB were less satisfied than AFAB. After performing a Bonferroni correction, differences on individual items did not remain significant. Chi-square tests showed that no significant sex differences were found for the threshold score of the PFSS either.

The GS-ASS was completed by the parents of 168 children. Univariate ANCOVA, with covariate age, did not show a significant effect of sex on total score of the GS-ASS. For individual items (Table 3), ANCOVA showed a significant sex × item 1 *(Does ___ say that (s)he would like a vagina/penis?; F*(1, 167) = 7.902, *p* = .001), sex × item 2 *(Does ___ say that (s)he would like breasts/no breasts?; F*(1, 166) = 6.181, *p* = .001) and sex × item 5 which remained significant after Bonferroni correction. AMAB scored higher on the items regarding gender-specific characteristics and were more satisfied. The fifth item (*Does ___ say that he/she wishes his face were prettier?*) differed significantly for birth assigned sex, where AMAB scored lower than AFAB (*F*(1, 167) = 8,596, *p* = .004). There was no difference between AFAB and AMAB in percentage scoring in the clinical range.

### Body image and intensity of GI

Multiple linear regression showed that intensity of GI measured with both the GIQ or GII had no significant relation with the outcome of the PAS, also when adjusted for birth assigned sex and age. When analyzing separately for birth assigned sex, no significant relations were found for either AMAB or AFAB.

Multiple linear regression showed that intensity of GI measured with the GII had a significant relation with outcome of the PFSS, also when adjusted for birth assigned sex and age (β = −.221, *t* = −2.205, *p* = .030). For AMAB, GII had a significant relation with the PFSS (β = −.308, *t* = −2.391, *p* = .021) only this disappeared when corrected for age. For AFAB there was no significant relation.

For intensity of GI measured by GIQ, linear regression showed no significant relation with PFSS, even when corrected for age and sex. When analyzing this relation separately for birth assigned sex, neither AMAB nor AFAB had a significant relation.

Multiple linear regression showed that the intensity of GI, measured by the GII, was significantly related to total score on the GS-ASS, also when corrected for age and sex (β = −.330, *t* = −8.543, *p* < .001). A higher total score on the GII was related to a lower total score on the GS-ASS. For both AMAB (β = −.334, *t* = −6.173, *p* < .001) and AFAB (β = −.322, *t* = −5.476, *p* < .001) the GII was significantly related to total score on the GS-ASS when corrected for age.

Multiple linear regression showed that the intensity of GI, measured by the GIQ, was also significantly related to total score on the GS-ASS, also when corrected for age and sex (β = 2.237, *t* = 5.564, *p* < .001). A higher mean score on the GIQ was related to a higher total score on the GS-ASS. A higher mean score on the GIQ was related to a higher total score on the GS-ASS. For both AMAB (β = 2.727, *t* = 4.110, *p* < .001) and AFAB (β = 1.724, *t* = 3.403, *p* = .001) the GII was significantly related to total score on the GS-ASS when corrected for age.

## Discussion

The current study aimed to provide a better understanding of body image in children referred for GI and examined the possible relation between body satisfaction and the intensity of GI. Our findings showed that a significant proportion of children with GI experienced body dissatisfaction. As expected, there were birth assigned sex differences for body satisfaction. AFAB reported more body dissatisfaction than AMAB. Furthermore, the intensity of GI showed to be related to dissatisfaction with gender specific body characteristics.

With regard to the findings in our study on the experience of body image, 4.1 - 49.4% of the children with GI experienced body dissatisfaction. First, it is important to note that not all children with GI suffer from a negative body image. Secondly, since body image was assessed with different measures, where the self-report measures focused on neutral body characteristics and the parent-reported measure focused on dissatisfaction towards gender specific body characteristics, the findings were somewhat variable.

On the parent-reported scale on gender-specific body dissatisfaction almost half of the group scored in the clinical range, meaning they have considerable dissatisfaction with their gender-specific characteristics. The percentage of children in the clinical range of dissatisfaction is much higher for this scale than for the two measures on neutral characteristics. Previous studies have also shown that adults with GI primarily report more body dissatisfaction with their primary and secondary sex characteristics, compared to their neutral body characteristics (Pauly & Lindgren, 1977; Van de Grift et al., 2016b). Although it may be a sensitive topic, we believe it is important to discuss body perception, and more specific, gender related body perception with children with GI to get a better understanding of how to support children with their feelings towards the body.

Based on the children’s self-reported measures, AFAB showed more body dissatisfaction of neutral characteristics than AMAB. This is consistent with findings of several previous studies on children who were not clinically referred for gender identity issues (Cole et al., 2001; Rauste-von Wright, 1988; Xanthopoulos et al., 2011). Gender differences in body dissatisfaction are well-established in the literature. AFAB in the general population tend to report more dissatisfaction than AMAB, both in childhood and over the course of adolescence into adulthood. The body dissatisfaction of AFAB is mainly due to greater sociocultural pressure to achieve a particular body type. Additionally, the social pressure may begin earlier for them than AMAB (Smolak, 2004). In this study, the AFAB were significantly older than the AMAB, therefore we hypothesize that social pressure influenced them the most. Combined with the given that puberty in AFAB can start at a younger age than AMAB (Parent et al., 2003), the AFAB were expected to have more body concerns and to experience more body dissatisfaction. Together this may account for the perceived differences between the sex assigned at birth groups.

Another important finding in our study is that the intensity of GI is related to the body satisfaction of children. As expected, a greater intensity of GI relates to more body dissatisfaction. For both AMAB and AFAB, intensity of GI is related to gender-specific body characteristics. In light of the future development of children with GI, the association found in our study between body satisfaction and the intensity of GI in children may be an important one. We know that not all children with GI become adolescents or adults with GI (Ristori & Steensma, 2016). We further know that besides age, birth assigned sex and social class of the parents, the intensity of GI is a strong predictor for a future gender incongruent development (Ristori & Steensma, 2016). However, body image has never been studied as a predicting factor in the development of children with GI. Our observation of the relation between body image and GI may be a strong argument to include body image in future studies and give us a better understanding of future development of children with GI.

Based on our findings, it is advised to keep the observed relation between body dissatisfaction and GI in mind while evaluating and counseling children with GI. Most measures used in child assessments have a focus on the behavioral- and social component of gender incongruence, while body image related questions seem to be more important when children grow into adolescence and puberty starts. Mental health practitioners should be aware of the diversity in body dissatisfaction in the group. The focus in counseling on this topic could be on helping the child to be more comfortable in discussing their body uneasiness. In addition to this, counseling should focus on helping the child to deal with their body dissatisfaction (and by no means focus on changing their gender identity or body image) and creating an open and safe environment for exploration. Counseling in this way is focused on supporting the child in their development.

### Suggestions for further research

To our knowledge, besides unpublished data, this is the first study focusing on body satisfaction of children with GI. Strengths of this study are the relatively large sample size and the fact that it was performed in the largest Dutch gender identity clinic, which treats more than 95% of the gender diverse population in the Netherlands (Wiepjes et al., 2018). As this study only focused on clinically referred children with GI, it would be informative if further studies also include non-referred children with GI. Furthermore, comparison studies with a control group of non-referred children are necessary. Additionally, as it is unclear whether body dissatisfaction experienced by children with GI is similar to that experienced by children without GI (Peterson et al., 2017), qualitative studies could provide us with more insight as to how children with GI experience discomfort with their body.

Both self-report and parent-report measures of body satisfaction were used in this study. This was both a strength and a limitation. Due to the different content of the questionnaires no comparisons between parent- and self-report could be made. Previous studies, however, showed that parents of children with GI reported similarly as their children on multiple questionnaires, including those focused on child body image (Durwood et al., 2017; Gilliland et al., 2007). Parent-report measures can differ from self-report child measures, especially concerning sensitive topics such as body image. In future research, it would be helpful if there was a self-report questionnaire on gender-specific body characteristics for children.

This study contained a diverse population with an age range up to the age of 12 years. Therefore, it may be possible that puberty had already begun in some of the older children, especially AFAB, during the diagnostic phase (Parent et al., 2003). Age and puberty could have an effect on body perception and satisfaction, and could therefore have influenced the findings. Adjustments for age were made in all analyses to control for this. Consequently, the results of this study were not likely to have been caused by age differences. However, it is important to note that start of puberty was not measured and was therefore not taken into account when analyzing these findings. It is a possibility that puberty may have already started in some of the children in this sample, which could have led to greater body dissatisfaction in AFAB.

There are many factors that influence body image for which no corrections were made in this study. Known factors related to body image in adults are culture, sexuality, activities, body mass index and psychopathology (Grogan, 2017; Kostanski & Gullone, 1998; Xanthopoulos et al., 2011). Important to note is that 8% of Dutch children with GI are diagnosed with an autism spectrum disorder, compared to 0.6% of the general child population (De Vries et al., 2010; Herremans et al., 2012). There are indications that autism spectrum disorder can influence how the body is experienced and evaluated (Krumm et al., 2017). Whether this applied to children should be further researched. Furthermore, other psychopathology diagnoses are also common in people with GD, which can increase risk for negative body image (Buddeberg-Fischer et al., 1999; Kostanski & Gullone, 1998; Lopez et al., 2016). Future studies on the body image of children with GI, should take these factors into account (Xanthopoulos et al., 2011).

A previous study by Johnson et al. found that the majority of children with GI never talks about their sexual anatomy (Johnson et al., 2004). The GS-ASS examines whether parents have heard their child express dislike of their gender specific characteristics. No questions about primary sex characteristics were asked of the children. As children rarely express dislike about their sexual anatomy, it is possible that children dislike their sexual anatomy but never express this to their parents. Therefore, the findings of the GS-ASS could be an underestimation. Still, the dissatisfaction found on the GS-ASS is significantly higher than the other measures of body image.

## Conclusion

Body dissatisfaction is observed in a significant proportion of children with GI. Therefore clinicians should be aware to focus on this topic in evaluation and counseling of children. Interventions to improve or make children more comfortable in their bodies should be considered. The observed relation between body dissatisfaction and GI seems to be an important finding with regard to the development of children with GI where body image should be taken into account as a predictor in the development of children with GI in future studies.

## Appendix A

### Physical Attractiveness Scale (PAS)

Directions: Look in the mirror and rate how much you agree with each statement about yourself from 0 (not at all) to 4 (a lot).

## Appendix B

### Physical Features Satisfaction Scale (PFSS)

Directions: Rate how much you like each of your own body parts from 0 (not at all) to 4 (a lot).

## Appendix C

### Gender Specific Anatomic Satisfaction Scale: Parent Report (GS-ASS; Lambert, 2009). Adapted

Directions: The following questions are about your child now or within the past year. Please answer each question by checking YES if you think your child is like this, NO, if your child is not like this, or SOMETIMES if you think your child is somewhat like this.

**Table:**

		Yes	Sometimes	No
1	Does ___ say that he would like a vagina? Does ___ say that she would like a penis?			
2	Does ___ say that he would like breasts? Does ___ say that she doesn’t want breasts?			
3	Does ___ say that he wished he looked like a girl? Does ___ say that she wished she looked like a boy?			
4	Does ___ say that he wishes to get rid of his penis? Does ___ say that she wishes to get rid of her vagina?			
5	Does ___ say that he wishes his face were prettier? Does ___ say that she wishes her face were prettier?			
6	Does ___ pretend that he has breasts? Does ___ pretend that she has a penis?			

## Notes

1 We decided to refer to the children in this study as birth assigned boys and girls. We are aware this is not their strongly held, firm gender identity and acknowledge that for a number of children this might not be how they identify themselves at a later age.
